# From Clinical Standards to Translating Next-Generation Sequencing Research into Patient Care Improvement for Hepatobiliary and Pancreatic Cancers

**DOI:** 10.3390/ijms18010180

**Published:** 2017-01-18

**Authors:** Ioannis D. Kyrochristos, Georgios K. Glantzounis, Demosthenes E. Ziogas, Ioannis Gizas, Dimitrios Schizas, Efstathios G. Lykoudis, Evangelos Felekouras, Anastasios Machairas, Christos Katsios, Theodoros Liakakos, William C. Cho, Dimitrios H. Roukos

**Affiliations:** 1 Centre for Biosystems and Genome Network Medicine, Ioannina University, 45110 Ioannina, Greece; ikyrochristos@hotmail.com (I.D.K.); deziogas@hotmail.com (D.E.Z.); 2 Department of Surgery, Ioannina University Hospital, 45110 Ioannina, Greece; gglantzounis@gmail.com (G.K.G.); chkatsios@gmail.com (C.K.); 3 Department of Surgery, ‘G. Hatzikosta’ General Hospital, 45001 Ioannina, Greece; 4 General Hospital of Grevena, 51100 Grevena, Greece; gizas24@yahoo.gr; 5 1st Department of Surgery, Laikon General Hospital, National and Kapodistrian University of Athens, 11527 Athens, Greece; schizasad@gmail.com (D.S.); evangelosf@hotmail.com (E.F.); theodlia@otenet.gr (T.L.); 6 Department of Plastic Surgery, Ioannina University School of Medicine, 45110 Ioannina, Greece; elykoudi@cc.uoi.gr; 7 Third Department of Surgery, Attikon General Hospital, Medical School, National and Kapodistrian University of Athens, 12462 Athens, Greece; anmach@med.uoa.gr; 8 Department of Clinical Oncology, Queen Elizabeth Hospital, Kowloon, Hong Kong, China; chocs@ha.org.hk; 9 Biomedical Research Foundation of the Academy of Athens (BRFAA), 11527 Athens, Greece

**Keywords:** next-generation sequencing (NGS), clinical implications, hepatobiliary and pancreatic (HBP) cancers

## Abstract

Hepatobiliary and pancreatic (HBP) cancers are associated with high cancer-related death rates. Surgery aiming for complete tumor resection (R0) remains the cornerstone of the treatment for HBP cancers. The current progress in the adjuvant treatment is quite slow, with gemcitabine chemotherapy available only for pancreatic ductal adenocarcinoma (PDA). In the advanced and metastatic setting, only two targeted drugs have been approved by the Food & Drug Administration (FDA), which are sorafenib for hepatocellular carcinoma and erlotinib for PDA. It is a pity that multiple Phase III randomized control trials testing the efficacy of targeted agents have negative results. Failure in the development of effective drugs probably reflects the poor understanding of genome-wide alterations and molecular mechanisms orchestrating therapeutic resistance and recurrence. In the post-ENCODE (Encyclopedia of DNA Elements) era, cancer is referred to as a highly heterogeneous and systemic disease of the genome. The unprecedented potential of next-generation sequencing (NGS) technologies to accurately identify genetic and genomic variations has attracted major research and clinical interest. The applications of NGS include targeted NGS with potential clinical implications, while whole-exome and whole-genome sequencing focus on the discovery of both novel cancer driver genes and therapeutic targets. These advances dictate new designs for clinical trials to validate biomarkers and drugs. This review discusses the findings of available NGS studies on HBP cancers and the limitations of genome sequencing analysis to translate genome-based biomarkers and drugs into patient care in the clinic.

## 1. Introduction

The integration of next-generation sequencing (NGS) technologies [1] and systems biology methods into the ENCODE project (Encyclopedia of DNA Elements) [2,3] has revolutionized biomedical research. New knowledge on non-coding genome functionality and genome-wide sequence variation have changed researchers’ thinking in the way health and disease are affected. A tremendous patient-centric research has began to transform medicine from an inexact science into precision medicine [4]. Genome-based directions in cancer research are urgently needed [5], as survival remains poor [6,7] either for the more advanced stages of major cancer types or even in some early and localized aggressive cancers.

Hepatobiliary and pancreatic (HBP) adenocarcinomas are considered to be among the most aggressive cancer types, as reflected by the clinical evidence of dismal prognosis [6,7]. High expectations for overcoming substantial therapeutic resistance, recurrence and cancer-related deaths rise from NGS applications. Accuracy, dropping costs, and speed have led to an explosion of NGS use in biomedical research over the past decade [8]. Accumulating evidence from early NGS studies provides promising results for identifying extensive genetic and genomic heterogeneity [9,10], which in turn suggests the need for the development of genome-based robust biomarkers and effective targeted drugs to improve personalized cancer medicine. However, multiple challenges have emerged, including the questionable validity of bioinformatics and the large sample sizes required to achieve statistical significance in the discovery of both novel cancer driver genes and therapeutic targets.

This review discusses the advances of modern surgery and oncology and, at the same time, describes the hurdles in improving oncological outcomes of HBP cancers. These tumors have been very difficult to effectively treat and are associated with high therapeutic resistance and relapse rates. Furthermore, we elaborate on the potential and limitations of NGS analysis to develop a biomarker-based patient selection system for more effective therapies, either with available targeted drugs or by discovering new agents targeting novel druggable mutations.

A search of the published literature was conducted in the PubMed database, using the following algorithm: (targeted OR tNGS OR whole-exome OR WES OR whole-genome OR WGS) AND next-generation sequencing AND (hepatocellular carcinoma OR hepatocellular cancer OR liver cancer OR cholangiocarcinoma OR bile duct cancer OR gallbladder cancer OR pancreatic cancer OR pancreatic adenocarcinoma).

## 2. Clinical Standards

The main cancer types comprising the HBP cancer group are the hepatocellular carcinoma (HCC), the intrahepatic and extrahepatic bile duct cholangiocarcinoma (ICC and ECC), gallbladder cancer (GBC), and the pancreatic ductal adenocarcinoma (PDA). Although incidence rates are relatively low for HBP cancers, mortality rates are very high, indicating the aggressiveness of these tumors. More specifically, primary liver cancers stand for 39,230 and 782,451 cancer cases annually in the US and worldwide, respectively, while they are responsible for 27,170 and 745,533 deaths in those areas. Similarly, 53,070 and 337,872 patients are diagnosed with pancreatic cancer each year in the US and globally, while 41,780 and 330,391 people die, respectively [6,7,11].

Sadly, screening is available only for patients at high risk for HCC. This group includes patients with hepatic cirrhosis, mainly due to hepatitis B or C, alcoholic and non-alcoholic fatty liver disease (NAFLD), as well as hepatitis B carriers, even without cirrhosis. The screening process includes the use of ultrasonography every six months. The combination with Alpha-fetoprotein (AFP) is not recommended, as the 6%–8% gain in the detection rate does not counterbalance the increase in false positive results, ultimately leading to an approximately 80% increase in the cost of each small HCC diagnosed (EASL–EORTC clinical practice guidelines 2012) [12].

Advances in cancer imaging have led to more accurate diagnosis of HBP cancers so that, unlike many other cancers, imaging remains the primary method for diagnosing and staging HBP cancers, and resection of those tumors usually does not require a biopsy. For example, diagnosis of PDA requires the use of Multi-Detector Computed Tomography (MDCT) angiography and the acquisition of thin, axial sections using a dual-phase pancreatic protocol [13]. The diagnosis of HCC requires the employment of a triple-phase CT (or MRI—Magnetic Resonance Imaging) scan with intravenous contrast and the presence of the radiological hallmark for HCC, i.e., contrast uptake in the arterial phase and washout in the venous/late phase. Non-invasive diagnosis is established by one imaging technique in nodules above 2 cm, showing the HCC radiological hallmark and two coincidental techniques with nodules of 1–2 cm in diameter in suboptimal settings (CT, MRI) [12,14]. The only indications for conducting a pre-surgery biopsy are for hepatic tumors that fail to produce the radiological hallmark of HCC (core biopsy is preferred over FNA—fine-needle aspiration), and always before starting a chemotherapy regimen. This last category includes borderline pancreatic adenocarcinomas that require neo-adjuvant treatment, and unresectable and metastatic tumors. Finally, diagnosis of biliary tract tumors is similarly based on imaging and, sometimes, on biopsy performed during the investigation of a hepatic mass.

Pre-surgery staging of HBP cancers aims at defining the resectability of the tumor and is based upon history and physical examination along with a number of imaging and laboratory studies, dependable on the specific type of cancer. For instance, the staging process of PDA additionally employs the use of chest imaging (CT preferred over X-rays), endoscopic ultrasound (EUS), MRI/magnetic resonance cholangiopancreatography (MRCP)/endoscopic retrograde cholangiopancreatography (ERCP), and liver function tests. FNA cytology during the EUS is performed when clinically indicated [15,16,17,18]. Staging of HCC consists of a hepatitis panel, serum levels of bilirubin, transaminases, alkaline phosphatase, albumin, creatinine and AFP, calculation of blood urea nitrogen (BUN) and international normalized ratio (INR), a complete blood count, a chest CT, and a bone scan if clinically indicated [19,20,21]. Moreover, preoperative volumetric assessment of total liver volume (TLV) and functional liver remnant (FLR) is mandatory in order to assess the minimum FLR (40% in chronic liver disease) that can sustain proper liver function. In addition, many centers, mainly in Asia, use the percentage of Indocyanine Green Clearance in 15 min (ICGR15, %) which is a very good marker of hepatic functional reserve and provides more information versus estimation of FLR alone [22]. Staging of biliary tract tumors includes CT or MRI scans with intravenous contrast, a chest CT, liver function tests, serum carcinoembryonic antigen (CEA), cancer antigen 19–9 (CA 19–9), and AFP levels, a hepatitis panel, biopsy and esophagogastroduodenoscopy, and colonoscopy, in the case of ICC. All decisions about resectability should be discussed at multidisciplinary oncologic meetings [23,24].

Complete surgical resection (R0, AJCC/UICC) [25,26], when feasible, remains the only potentially curative option for treating HBP cancers. Thus, the primary treatment option for resectable HCC tumors is the partial hepatectomy [27,28]. Furthermore, patients meeting the United Network for Organ Sharing (UNOS) [29] criteria can undergo a liver transplantation, addressing both the issues of HCC and possible underlying chronic liver disease. For GBC, a cholesystectomy must be followed by en bloc hepatectomy (removal of segments IVb and V) and regional lymphadenectomy with or without bile duct excision, except for T1a tumors incidentally found on pathologic review after cholesystectomy, for which observation is advised [30]. Lymph node excision is also advised after resection of ICC for accurate post-operative staging [31]. Lastly, the type of surgical procedure executed for resectable pancreatic tumors depends on the location and tumor-node-metastasis (TNM) status of the PDA. The procedures include the pancreatoduodenectomy (Whipple procedure) and the distal pancreatectomy with splenectomy, often en bloc [23,24,32].

### 2.1. Modern Adjuvant and Neoadjuvant Treatment

In contrast to the majority of cancers adjuvant therapy of any kind is not advised in the case of HCC [24,33,34,35]. Adjuvant treatment of GBC includes fluoropyrimidine chemoradiation (except T1a or T1b, N0) and fluoropyrimidine- or gemcitabine-based chemotherapy, although observation is not ill-advised [36,37]. Adjuvant therapy for ICC depends on the post-resection status, as to the presence of residual disease. After R0 resection, fluoropyrimidine- or gemcitabine-based chemotherapy is advised equally to simple observation or participation in a clinical trial. R1 resection or regional node involvement requires chemotherapy (fluoropyrimidine- or gemcitabine-based) or fluoropyrimidine chemoradiation. Lastly, R2 resection calls for one of the following: combination therapy with gemcitabine and cisplatin, a chemotherapy regimen as above, participation in a clinical trial, or locoregional therapy, always with the best possible supportive care [36,37,38,39]. In ECC patients, adjuvant treatment again is based on the post-resection status. For R0 resections with negative regional nodes or for in situ carcinomas, the patient can simply be observed or undergo an adjuvant treatment regimen among fluoropyrimidine chemoradiation, fluoropyrimidine- or gemcitabine-based chemotherapy or participate in a clinical trial. After R1 or R2 resection, it is advised that patients further be subjected to chemoradiation followed by chemotherapy. As for regional lymph node involvement, adjuvant treatment consists of chemotherapy alone [36,37,38].

PDAs are staged preoperatively as resectable, borderline resectable and unresectable, based on tumor contact with specific blood vessels [13]. Borderline resectable PDA is the only HBP cancer for which neo-adjuvant systemic treatment is advised, followed by imaging studies to assess respectability. Acceptable neoadjuvant regimens include FOLFIRINOX, gemcitabine plus albumin bound paclitaxel, and chemoradiation (fluoropyrimidine- or gemcitabine-based), alone or after chemotherapy [40,41,42,43,44]. Adjuvant treatment depends on whether the patient received neoadjuvant therapy or not. In the latter case, recommendations regarding adjuvant treatment include participation in a clinical trial, chemoradiation (fluoropyrimidine- or gemcitabine-based) preceded and followed by chemotherapy (gemcitabine or 5-FU/leucovorin or continuous infusion of 5-FU), or chemotherapy alone [45,46,47,48]. On the other hand, patients who received prior neoadjuvant treatment are advised to consider additional chemotherapy. In general, due to the lack of large scale randomized controlled trials (RCTs) about adjuvant and neoadjuvant treatment options against HBP cancers, patients are encouraged to participate in clinical trials [23,24].

Treatment of unresectable and metastatic HBP carcinomas is mainly based on systemic chemotherapy. In HCC, treatment options for intrahepatic unresectable disease include locoregional therapy (trans-arterial chemoembolization), systemic targeted therapy with sorafenib in advanced HCCs with macrovascular invasion, or some other chemotherapy regimen. Metastatic HCCs are also treated with sorafenib [49,50,51,52]. Sorafenib is an inhibitor of several tyrosine protein kinases, such as vascular endothelial growth factor receptors (*VEGFR*), platelet-derived growth factor receptor (*PDGFR*), and Raf family kinases (more avidly *C-Raf* than *BRAF*) [53,54]. It is indicated for the treatment of advanced renal cell carcinoma, advanced hepatocellular carcinoma, and radioactive iodine-resistant advanced thyroid carcinoma, without biomarker-based patient selection [51,54,55,56]. It is currently the first of only two targeted drugs used against HBP cancers, the other being erlotinib. Treatment for unresectable and metastatic biliary tract cancers consists of combination therapy with gemcitabine and cisplatin, or another chemotherapy regimen based on gemcitabine or fluoropyrimidine. Unresectable, non-metastatic bile duct tumors can also be treated with fluoropyrimidine chemoradiation [37,38,39].

Management of unresectable and metastatic PDAs depends upon the patient performance status (PS). If PS is good, therapeutic options include the FOLFIRINOX regimen, gemcitabine alone, combined with albumin-bound paclitaxel or with erlotinib, or another gemcitabine-based combination therapy. In the case that PS is poor, there is only the option of gemcitabine monotherapy and palliative care. Erlotinib hydrochloride is a drug approved for the treatment of non-small cell lung cancer (NSCLC) and advanced pancreatic cancer. It is a tyrosine kinase inhibitor, which acts on the epidermal growth factor receptor (*EGFR*) and is the second targeted therapy against HBP cancers, along with sorafenib. Patients with locally advanced, unresectable tumors can also be treated with chemoradiation (fluoropyrimidine- or gemcitabine-based), preferably after a course of chemotherapy [40,41,57,58,59,60,61]. All patients with unresectable and metastatic HBP cancers must be provided with the best possible supportive care and are encouraged to participate in clinical trials [23,24].

### 2.2. Limitations of Current Therapeutic Interventions

**Figure 1:**
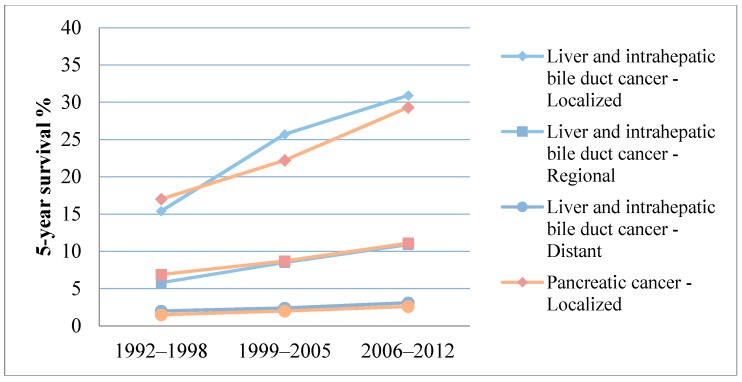
Five-year survival rates for hepatobiliary and pancreatic (HBP) cancers by stage from 1992 to 2012 [7,63,64].

Despite all this progress in the diagnosis and treatment of HBP cancers, 5-year survival rates (5-YSR) remain disappointing, even for early, localized tumors, as we can see in Table 1 [7,62]. Even though some improvement can be seen over the past two decades, particularly for early stage tumors, all stages of HBP cancer still feature very poor survival (Figure 1) [7,63,64]. For instance, combined 5-YSR for localized liver, intrahepatic bile duct, and pancreatic cancer is about 30%, which is minimal when compared to 5-YSR of over 90% for localized breast and colorectal cancer [6,7]. Nevertheless, specialized, high-volume HBP centers have recently reported improved 5-YSR of 45%–56% for HCC [22,65]. These numbers can be explained by the very high rates of tumor recurrence after complete resection that range from 54% for HCC [65] to 81% for PDA [48]. Because the majority of recurrences after hepatic resection (66%) [65] are limited to the liver, overall recurrence rates are as low as less than 20% after liver transplantation [66]. Recurrence rates in further detail are demonstrated in Table 2 [48,65,66,67,68,69,70,71,72,73,74,75,76,77,78,79,80]. HCC incidence can be decreased either by mass vaccination programs against hepatitis B virus or by adequate antiviral treatment.

High recurrence and low survival rates have led to a very large number of clinical trials concerning systemic treatment of HBP cancers, and medical interest has focused on the development of new targeted drugs. Targeted therapy is the most recent major modality of treatment for cancer, along with cytotoxic chemotherapy and hormonal therapy. Targeted drugs block tumor growth by interfering with specific molecules vital to carcinogenesis, rather than harming all rapidly dividing cells, thus causing fewer and less intense adverse effects compared to chemotherapy. Sadly, only approximately 20 Phase III randomized controlled trials (RCTs) on targeted drugs against HBP cancers have reached completion. Until now nearly all completed Phase III RCTs for HBP tumors feature negative or not statistically significant results, as we can see in Table 3 and Table 4 [81,82,83,84,85,86,87,88,89,90,91,92,93,94,95,96,97,98,99]. By contrast, two studies have reported promising results. The RESORCE trial, which examines regorafenib as a second line treatment after sorafenib, has featured a significantly increased overall survival of 10.6 months versus 7.8 months for the placebo (hazard ratio—HR = 0.62 [95% confidence interval—CI, 0.50–0.78]; *p* < 0.001) on the preliminary report [100]. A second and most impressive example is a study by Lee JH et al. that evaluates the efficacy of autologous cytokine-induced killer cells in the adjuvant setting after a complete resection of HCC, that featured a significant overall survival benefit (HR = 0.21 [95% CI, 0.06–0.75]; *p* = 0.008), although it has not yet achieved FDA approval and has not been included in current guidelines [23,24,89].

### 2.3. Underway Phase III RCTs

Approximately 20 Phase III RCTs on targeted drugs are underway but only four are using biomarker-based patient selection. The REACH-2 trial investigates the effects of ramucirumab as second line treatment after sorafenib on patients with advanced HCC and elevated baseline AFP ≥ 400 ng/mL, after observing favorable effects of ramucirumab only on patients of this subgroup in the REACH trial, while the overall cohort of the study was negative [86,101]. The METIV-HCC trial uses tivantinib as second line treatment of MET-high, inoperable HCC tumors, after correlation with longer time to progression on a Phase II clinical trial [102,103]. The third trial (NCT02395016) uses nimotumumab in addition to gemcitabine on patients with *K-RAS* wild-type, advanced PDA. Lastly, the POLO trial examines the effects of olaparib on patients with germline *BRCA1/2* mutated PDA, who have shown no progression on first line platinum-based chemotherapy [104].

It becomes obvious that, despite the great interest in targeted therapy on behalf of pharmaceutical companies, most large-scale RCTs with hundreds of patients enrolled end up with negative results. This underlines the urgent need for an early drug development strategy, in order to predict drug efficacy and the capability of FDA approval [105]. The recent evidence on substantial genetic and genomic tumor heterogeneity by NGS has shifted biomedical interest to the research for the development of robust biomarkers and effective targeted drug discovery.

## 3. NEXT-Generation Sequencing and Tumor Heterogeneity

The advent and rapid progress of NGS technologies over the past decade has revolutionized biomedical research [1]. The unprecedented potential provided by NGS for accurate identification of genomic alterations (GAs) underlying common and rare diseases, has led to a tremendous effort in moving forward from current medicine as an inexact science and building the foundations of genomics-based precision medicine [4,8,106,107].

Particularly in cancer, definitive evidence for extensive genetic tumor heterogeneity [9] and validity of NGS technologies by a recent international consensus panel [108] have resulted in an explosion in NGS use on patient-derived sample analysis. Early after the introduction of NGS, fresh frozen sample collection and storage in biobanks was the standard procedure. However, this approach is time and resource consuming and is limited by the obligatory prospective design of clinical studies and the lack of long-term patient follow-up data. Technical developments have recently allowed the DNA/RNA extraction from formalin-fixed paraffin-embedded (FFPE) for NGS analysis, including targeted next-generation sequencing (tNGS), whole-exome sequencing (WES), and whole-genome sequencing (WGS), with similar accuracy as fresh frozen tissue sample NGS analysis [109,110].

Although clonal evolution and tumor heterogeneity have been proposed by Nowell in 1976 [111], evidence on genetic and genomic variation has emerged in the last two decades [112]. This tumor diversity can be crucial for developing more effective, personalized treatments for HBP cancers, to overcome current substantial therapeutic resistance and high recurrence rates of these tumors. For example, large-scale Phase III RCTs with a recruited population of over 1000 patients, testing the efficacy of targeted drugs (i.e., sorafenib in the adjuvant setting of HCC [81], linifanib as the first line in advanced HCC [82]) were negative. The failure of these studies to meet their endpoints can be explained by the empirical approach without either understanding the molecular landscape underlying therapeutic resistance or using biomarker-based patient selection for recruitment. Recent advances in NGS can overcome these limitations by implementing tNGS, WES, and WGS in new designs of clinical trials.

### 3.1. Targeted Next-Generation Sequencing

Over the past few years, the ability of NGS application on FFPE samples has led to the routine use of tNGS in public and private laboratories for screening a panel of genes to identify alterations matched to available targeted drugs [113,114]. Apart from decision-making on selecting an agent from the available FDA-approved list, tNGS provides the potential for performing new-design clinical trials, including umbrella and basket studies. For any specific cancer type (i.e., pancreatic cancer), the identification of subgroups of patients with similar druggable GAs could predict the positivity of umbrella clinical trials at an early stage. By contrast, basket design studies are performed to assess the clinical utility of specific targeted drugs on several different cancer types with similar GAs [105], such as transtuzumab for HER2-positive breast and gastric cancer [115]. However, tNGS has limited or no potential to identify novel cancer driver genes and therapeutic targets to overcome drug resistance.

Twenty targeted NGS studies were eligible for inclusion in the present review, studying HCC (Table 5) [116,117,118,119,120,121], biliary tract cancer (BTC) (Table 6) [122,123,124,125,126,127,128,129], and PDA (Table 7) [130,131,132,133,134,135].

Genetic heterogeneity between different cancer types was confirmed by the identification of distinct mutations in cancer driver genes, specific to each cancer. In a large study by Kawai-Kitahata et al. [121], the most frequently mutated gene in HCC tumors was *TERT* in 65% of the cases, and its mutational status was associated with HCV infection. tNGS studies also revealed potential utility in predicting HCC response to sorafenib [119,120]. Moreover, tNGS highlighted the genetic distinction between the different types of BTC [122,126,127,128]. One of the most frequently mutated and promising genes in BTC was *ARID1A*, mostly altered in ICC and GBC [129]. In addition, in ICC patients, identification of *FGFR* mutations may be of predictive value as to the therapeutic response to *FGFR* inhibitors, such as pazopanib and dovitinib [129]. Implementing tNGS in clinical trials for various cancer types could enhance predictive biomarker identification for improving drug efficacy [136]. Notably, a significant portion of BTC patients, ranging from 68% to 71%, carried potentially actionable gene alterations [123,128]. Concerning PDA, substantial mutational heterogeneity was discovered [133] and *KRAS* was the most commonly mutated gene [132]. A significant number of cases featured potentially targetable genes, such as *HER2* amplifications that are targeted by transtuzumab and wild-type *KRAS*, which could be an indication for administration of cetuximab, as in colorectal cancer [135].

Recent technical developments allowing NGS analysis on small amounts of tissue have enabled targeted sequencing of FNA samples in the diagnostic and neo-adjuvant setting broadening the clinical implications of NGS [137,138,139]. A recent study showed that tNGS could be performed on FNA samples with reliability comparable to that of conventional tumor samples, in order to achieve pre-treatment biomarker-based patient selection for clinical trials [132].

### 3.2. Whole-Exome and Whole-Genome Sequencing

Following the ENCODE project that explored the functionality of human genome [2], the exome (protein-coding area), accounting for 1.5% of the genome, can accurately be sequenced by WES. The remaining, non-coding genome that accounts for 98.5%, can only be sequenced by WGS.

Particularly in cancer, progress in NGS analysis of clinical samples is much faster for WES studies than for WGS, for which bioinformatics analysis on big data is highly complex and has not yet been standardized [108]. For example, the continuously dropping cost and the potential for identifying novel cancer driver genes has led to the largest WES study available, which has analyzed 4742 humans tumors for point mutations in the exome [9]. On the contrary, the largest WGS study available has analyzed 300 tumors [10]. Despite its complexity and much higher cost, WGS is essential for identifying large structural genome changes, such as copy number alterations (CNAs) and chromosomal rearrangements that have a critical role in cancer diagnosis and therapy. Moreover, based on the functionality of most of the non-coding genome (~80%), another strength of WGS is the ability to identify non-coding sequence variations. When WGS is coupled with RNA-sequencing (RNA-seq) and chromatin immunoprecipitation sequencing (ChIP-Seq), their combination provides the potential for transcriptome analysis and the future development of next-generation drugs that disrupt transcriptional biocircuits [140]. Despite the advances in WGS, many challenges have to be overcome, including high costs and a lack of bioinformatics standardization.

WES studies on HBP cancers, summarized in Table 8 [141,142,143,144,145,146,147,148,149,150,151,152,153,154,155], are consistent with the extensive genetic tumor heterogeneity identified by the largest WES study available on 21 other cancer types by Lawrence et al [9]. Fifteen WES studies were deemed eligible for inclusion in this review. Frequently mutated genes were *KRAS* in PDAs [146,151], chromatin regulation genes, and especially *ARID1A* in PDAs and HCCs [142,145,148,150,151,155] and *TERT* in 68% of HCCs [155]. Many genes and genomic alterations (GAs) with possible diagnostic, prognostic, and therapeutic significance were identified, including ten novel genes [143,149] and a recurrent novel fusion (*FGFR2*-*PPHLN1*) in 16% of ICCs [152]. Interestingly, a significant portion of HBP patients approximate a 30% share targetable or potentially targetable mutations in known genes [144,152,154], such as *FGFR*, which can be targeted by FGFR inhibitors [152] and *ARID1A*, which will be discussed in further detail below.

WGS studies provide powerful potential for the assessment of both the genetic and genomic heterogeneity, including CNAs and chromosomal rearrangements. Ten WGS reports on HBP cancers were included in our review, and their findings are outlined in Table 9 [10,155,156,157,158,159,160,161,162,163,164]. Recurrent mutations were identified in chromatin regulators and particularly *ARID1A* and *ARID2* in HCCs and PDAs [160,163,164] and in the *TERT* [10,155,160,162] and *TERT* promoter locus [163] in all kinds of HBP cancers. A number of cancer-related genes were highlighted, including well-known genes, as well as five novel genes [10,164]. Environmental risk factors, and especially viral hepatitis and alcohol, were associated with specific mutated genes in primary liver cancer, shaping potential clinical benefit [10,159,161,163]. Furthermore, one study compared the WGS analyses of two multicentric HCCs and concluded that the nodes originated from distinct, independent mutations [160].

### 3.3. Confirmation of Known Cancer Driver Genes by NGS Supporting Clinical Implications

Our review deemed two genes as noteworthy: *TERT* and *ARID1A*. *TERT* alterations were discovered mainly in HCC patients with tNGS [121], WES [155], and WGS [10,155,160,162,163]. On the other hand, *ARID1A* aberrations were uncovered in all types of HBP cancer by tNGS [129], WES [142,145,150,151,155], and WGS [155,160,164].

Telomerase reverse transcriptase (abbreviated to *TERT*, *hTRT*, or *hTERT* in humans) is a telomerase enzyme catalytic subunit, along with the telomerase RNA component (*hTR* or *TERC*). Telomerase is expressed, and telomere length is maintained in human germ cells and the vast majority of primary human cancers (~90%), deterring apoptosis [165,166]. Many cancers also exhibit mutations in the *TERT* promoter locus, which increase transcriptional activation of this gene [167]. Regulation of *TERT* and telomerase activity is achieved through a large number of mechanisms and complexes, including the mTOR pathway [166]. Therefore, *TERT* has been an important target for anticancer treatment, especially immunotherapy, in the past twenty years with mostly unfavorable results [167], despite the successful targeting of telomerase-positive tumor cells in in vitro and in mouse model studies [168].

The *ARID1A* gene in humans encodes the AT-rich interactive domain-containing protein 1A [169]. This protein is a member of the SWI/SNF family of proteins that are believed to regulate the transcription of certain genes by altering the chromatin structure [170,171,172]. The SWI/SNF complex has also been implicated with DNA repair mechanisms [173]. Several sequencing studies have identified SWI/SNF as a tumor suppressor in a number of diverse malignancies [174,175,176,177,178]. A meta-analysis of 24 whole-exome sequencing studies reported SWI/SNF to be mutated in approximately 20% of human malignancies and *ARID1A* to be the most commonly mutated gene of the complex [179]. *ARID1A* has commonly been found mutated in gastric cancer [180], ovarian clear cell carcinoma [178], hepatocellular carcinoma [160], and pancreatic cancer [164,175].

*ARID1A* is a promising potential target for future therapies. A recent study on ovarian clear cell carcinoma has demonstrated a synthetic lethality between *ARID1A* mutation and targeted inhibition of enhancer of zeste homolog 2 (*EZH2*) methyltransferase through upregulation of *PIK3IP1*, which is a negative regulator of *PI3K* [181,182]. Furthermore, *EZH2* inhibition (*EZH2i*) has been shown to block tumor formation driven by *SNF5* (member of the SWI/SNF complex) inactivation in rhabdoid tumors [183]. *EZH2i* was also found to alter the response to etoposide in patients with non-small-cell lung cancer, dependent on the mutational status of *BRG1* (another member of the SWI/SNF complex) [184]. These findings suggest that targeted *EZH2i* is a compelling strategy against cancers with mutated SWI/SNF complex and *EZH2* inhibitors are currently under clinical development with promising preclinical results [185]. Lastly, another study has identified *ARID1B*, an *ARID1A* homolog, as the most essential gene for the survival of *ARID1A*-mutant cancer cells. The loss of *ARID1B* in cells with silencing *ARID1A* mutations impairs cell proliferation, making it a potential therapeutic target in *ARID1A*-deficient tumors [186].

### 3.4. Inter-Patient Heterogeneity and Personalized Therapeutic Approach

The evidence of tumor heterogeneity and the clinical challenge of interpatient variation-based personalized treatment could most likely be enabled by WES and WGS studies [9,187]. Two international, large projects, The Cancer Genome Atlas (TCGA) [188] and the International Cancer Genome Consortium (ICGC) [189], aiming at completing the cancer driver genes and mutations catalogue for 50 cancer types using NGS platforms, have already identified many novel genes involved in tumorigenesis. The largest WES study available was conducted by Lawrence et al. [9] spanning 21 tumor types, including 12 from (TCGA) and 14 from non-TCGA projects at the Broad Institute, with some overlapping tumor types. Based on extensive tumor heterogeneity, Lawrence et al. recommend strict criteria for reporting novel cancer genes, such as strong statistical significance (*p* < 0.01), suggesting large numbers (>500) of tumor samples [9]. Therefore, the novel genes identified by WES (Table 8) and WGS (Table 9) with possible clinical utility as biomarkers or novel therapeutic targets [149,164] require confirmation by larger studies.

## 4. Future Perspectives

Despite intensive research effort, relapse rates of HBP cancers remain substantially high. There has been slow progress in the development of effective systemic therapies. Only a few therapeutic options are available, including sorafenib approved only for advanced-metastatic HCC, gemcitabine in the adjuvant and metastatic setting of PDA, and erlotinib for locally advanced or metastatic PDA. Moreover, recent, large-scale, negative Phase III RCTs testing the efficacy of targeted drugs (Table 3 and Table 4) suggest the urgent need to shift from inexact medicine to understanding and precisely targeting molecular mechanisms underlying therapeutic resistance. Conventional single biopsy-based NGS studies, by enabling the identification of novel cancer driver genes and druggable mutations, shape a new avenue in the development of more effective systemic therapeutic interventions. Based on the discovery of novel actionable alterations by NGS, the concept of an early drug-development strategy could significantly broaden the list of approved targeted drugs. Given the considerable interpatient heterogeneity, this approach could improve primary therapeutic decision-making. One of the greatest challenges will be the translation of cancer genome sequencing discoveries into patient care [190].

However, accumulating evidence of intratumor heterogeneity (ITH) of the primary cancer [191,192] and the dynamics of genomic subclones according to the principles of Darwinian evolution [193] limit the expectations for overcoming primary and particularly acquired therapeutic resistance with the simple concept of single-biopsy NGS analysis. Breakthrough methods of NGS applications, including multi-regional biopsies for genome sequencing to identify ITH and repeated NGS of plasma circulating cell-free DNA or circulating tumor DNA (cfDNA, ctDNA) can identify dynamic emergence of distinct subclones. By comparing the GA landscape of the primary tumor to that of circulating genomic subclones (CGS) and the relapsed tumors in individual patients, we could develop biomarkers to predict and monitor disease relapse [194]. Furthermore, an early targeting of the identified resistant subclones could overcome therapeutic resistance, prolonging time to recurrence or even preventing metastatic relapse [140].

## 5. Conclusions

The validity of NGS technologies to identify tumor heterogeneity-associated therapeutic resistance and relapse gives rise to high expectations for translating these advances into patient-centric trials and clinical benefit. In the medium term, tNGS enables the conduction of umbrella and basket clinical trials. The identification of mutated or amplified gene-based patient subgroups and the subsequent tumor-guided treatment with targeted drugs from the list of available FDA-approved agents, matching these specific genetic alterations, could improve personalized patient care.

By contrast, the discovery of novel therapeutic targets by WES and WGS studies raises much higher expectations to substantially broaden the targeted drugs catalogue with a long-term perspective. However, this concept requires evaluation and confirmation by appropriately designed large-scale clinical trials. Therefore, tNGS, WES, and WGS could enable the development of robust biomarkers for tailored treatment.

In summary, translational NGS research represents a top prospect for faster progress than any other available technology to achieve precision oncology.

## Figures and Tables

**Table 1:** Five-year survival rates for hepatobiliary and pancreatic (HBP) cancers.

Cancer Type	Localized	Regional	Distant	Unstaged	Overall
Liver and intahepatic bile duct cancer	30.9% [7]	10.9% [7]	3.1% [7]	6.1% [7]	17.5% [7]
Intrahepatic bile duct cancer	15% [62]	6% [62]	2% [62]	N/A	N/A
Extrahepatic bile duct cancer	30% [62]	24% [62]	2% [62]	N/A	N/A
Pancreatic cancer	29.3% [7]	11.1% [7]	2.6% [7]	4.9% [7]	7.7% [7]

N/A: Not available.

**Table 2:** Recurrence rates of hepatobiliary and pancreatic (HBP) cancers after surgical resection, in Phase III randomized controlled trials (RCTs) or retrospective studies.

Type of Cancer	Treatment	Recurrence Rate (%)
HCC	Hepatectomy	51% [67]; 54% [65]; 63% [68]
HCC	OLT	7.6% [69]; 15.3% [66]; 18.3% [70]
PDA	Pancreatectomy and adjuvant chemotherapy	75%–85% [71]; 81%–93% (Phase III RCT) [48]
ICC	Resection	62.2% [72]; 70% [73]
Mixed HCC-CC/ICC Hilar/ICC	OLT	60% [74]; 38% [75]
ECC		
Distal	Resection	39% [76]
Hilar	Resection, OLT	53% [77]; 68% [78]; After OLT: 20% [79]
GBC	Resection, resection and adjuvant chemotherapy	66% [78]; 81.4% [80]

HCC: hepatocellular carcinoma; OLT: orthotopic liver tranplantation; PDA: pancreatic ductal adenocarcinoma; ICC: intrahepatic bile duct cholangiocarcinoma; HCC-CC: hepatocellular cholangiocarcinoma; ECC: extrahepatic bile duct cholangiocarcinoma; GBC: gallbladder cancer.

**Table 3:** Completed Phase III RCTs on targeted drugs for hepatocellular and bile duct cancers.

*N*	Setting	Intervention	Results	Reference/Clinicaltrials.gov Identifier
1114	Adjuvant HCC	Sorafenib vs. Placebo	RFS HR = 0.940; [95% CI, 0.780–1134]; one-sided *p* = 0.26	STORM trial [81]
1075	Advanced HCC First-line	Sunitinib vs. Sorafenib	Terminated based on a higher incidence of serious adverse events in the sunitinib and on failure to demonstrate superiority or non-inferiority to sorafenib	NCT00699374
1035	Advanced HCC First-line	Linifanib vs. Sorafenib	OS HR = 1.046; [95% CI, 0.896–1.221]	[82]
870	Intermediate Unresectable HCC	Brivanib vs. Placebo after TACE	HR = 0.90 [95% CI, 0.66–1.23]; log-rank *p* = 0.5280	[83]
720	Advanced HCC First-line	Sorafenib + Erlotinib vs. Sorafenib + Placebo	OS 9.5 vs. 8.5 months, HR = 0.929; *p* = 0.408	SEARCH trial [84]
635	Advanced HCC Second-line	ADI-PEG 20 vs. Placebo	OS 7.8 vs. 7.4 months; HR = 1.022 [95% CI, 0.847–1.233]; *p* = 0.884 PFS 2.6 vs. 2.6 months; HR = 1.175 [95% CI, 0.964–1.432]; *p* = 0.075	[85]
565	Advanced HCC Second-line	Ramucirumab vs. Placebo after Sorafenib	9.2 vs. 7.6 months; HR = 0.87 [95% CI, 0.72–1.05]; *p* = 0.14 HR = 0.674; *p* = 0.0059 with baseline AFP ≥ 400 ng/mL	REACH trial [86]
420	Advanced HCC	Tamoxifen + SOC vs. SOC alone	OS 4.8 [95% CI, 3.6–6] vs. 4.0 months [95% CI, 3.5–4.5]	[87]
395	Advanced HCC Second-line	Brivanib vs. Placebo	OS 9.4 vs. 8.2 months; HR = 0.89 [95.8% CI, 0.69–1.15]; *p* = 0.3307	BRISK PS trial [88]
230	Adjuvant HCC	CIK vs. Placebo	RFS 44.0 vs. 30.0 months; HR = 0.63; [95% CI, 0.43–0.94]; *p* = 0.010 OS HR = 0.21 [95% CI, 0.06–0.75]; *p* = 0.008	[89]
124 *	Advanced BDC	Cis/Gem + Cediranib vs. Cis/Gem + Placebo	PFS HR = 0.93 [95% CI, 0.65–1.35]; *p* = 0.72	ABC-03 trial [90]

Bile duct cancer (BDC); hazard ratio (HR); hepatocellular carcinoma (HCC); overall survival (OS); progression-free survival (PFS); recurrence-free survival (RFS); standard of care (SOC); trans-arterial chemoembolization (TACE); * Phase 2/3 RCT.

**Table 4:** Completed Phase III RCTs on targeted drugs for pancreatic ductal adenocarcinoma.

*N*	Setting	Intervention	Results	Reference
1062	Advanced PDA First-line	Arm I: Chemotherapy alone Arm II: Chemotherapy with sequential GV1001 (telomerase peptide vaccine) Arm III: Chemotherapy with concurrent GV1001	Sequential chemoimmunotherapy group OS HR = 1.19 [98.25% CI, 0.97–1.48]; *p* = 0.05 Concurrent chemoimmunotherapy group OS HR = 1.05 [98.25% CI, 0.85–1.29]; *p* = 0.64	TeloVac trial [91]
745	Locally Advanced PDA First-line	Gemcitabine + Cetuximab vs. Gemcitabine alone	OS HR = 1.06 [95% CI, 0.91–1.23]; *p* = 0.23, one-sided	Southwest Oncology Group-directed intergroup trial S0205 [92]
722	Adjuvant PDA	Algenpantucel-L (HAPa) Immunotherapy + SOC vs. SOC alone	Study completed No statistically significant difference on preliminary report OS 27.3 vs. 30.4 months	IMPRESS trial [93]
688	Advanced PDA First-line	Gemcitabine + Tipifarnib (R115777) vs. Gemcitabine + Placebo	OS HR = 1.03 [95% CI, 0.86–1.23]; stratified log-rank *p* = 0.75	[94]
632	Advanced PDA First-line	Gemcitabine + AG-013736 (Axitinib) vs. Gemcitabine + Placebo	OS HR = 1.014 [95% CI, 0.786–1.309]; *p* = 0.5436	[95]
602	Advanced PDA First-line	Gemcitabine + Bevacizumab vs. Gemcitabine plus Placebo	OS HR = 1.044 [95% CI, 0.88 to 1.24]; *p* = 0.95	CALGB 80303 trial [96]
160 *	Metastatic PDA First-line	Rigosertib (ON 01910.Na) + Gemcitabine vs. Gemcitabine alone	OS HR = 1.24 [95% CI, 0.85–1.81]	[97]
154	Advanced PDA First-line	G17DT immunogen vs. Placebo	Mortality HR = 0.75 [95% CI, 0.51–1.10]; *p* = 0.138	[98]
153 *	Advanced PDA First-line	Elpamotide + Gemcitabine vs. Placebo + Gemcitabine	OS HR = 0.87 [95% CI, 0.486–1.557]; Harrington-Fleming *p*-value = 0.918; log-rank *p*-value = 0.897	[99]

Hazard ratio (HR); overall survival (OS); pancreatic ductal adenocarcinoma (PDA); * Phase 2/3 RCT.

**Table 5:** Targeted next-generation sequencing studies on hepatocellular carcinoma.

*N*	Findings	Clinical Implications	Reference
9 tNGS; 1 WES	Mutations were observed in *TP53* and *CTNNB1* genes in 5/9 tumors	Larger studies are required	[116]
12	*TP53* mutations in 5/12 patients, *RUNX1* in 3/12 and other less frequent mutations	Larger studies required	[117]
14 (advanced-metastatic)	Mutations identified in several well-known genes and pathways	Larger studies required	[118]
45 patients (pts) treated with sorafenib (tNGS and CN assay; 6 CR, 39 non-CR)	*FGFR* mutations in 5/45 *FGF19* copy number gain was detected more frequently among CR cases (2/6 vs. 2/39; *p* = 0.024)	Larger studies are required to evaluate potential clinical utility of CN gain for *FGF19* as a predictive biomarker to sorafenib	[119]
46 pts treated with sorafenib	Average number of detected oncogene mutations differed significantly between the PD and non-PD groups (*p* = 0.0446)	Targeted sequencing could predict response to sorafenib	[120]
104	Most frequent mutations: *TERT* (65%, associated with HCV infection), *TP53* (38%, associated with HBV infection), *CTNNB1* (30%, associated with absence of HBV infection)	*TERT* promoter mutations are related to poor prognosis Results may influence diagnostic and therapeutic strategies	[121]

Complete response (CR); copy number (CN); progressive disease (PD); targeted next-generation sequencing (tNGS); whole-exome sequencing (WES).

**Table 6:** Targeted next-generation sequencing studies on biliary tract cancers.

*N*	Findings	Clinical Implications	Reference
11 (3 ICC, 8 ECC)	Molecular heterogeneity was identified between ICC and ECC	This molecular classification could potentially provide personalized therapeutic implications	[122]
28	In 71% of cases, at least one potentially actionable alteration was found in known genes	Identification of these novel gene fusions (*FGFR2-KIAA1598, FGFR2-BICC1, FGFR2-TACC3*, and *RABGAP1L-NTRK1*) provides potential for personalized treatment	[123]
40 (15 ECC, 10 ICC, 14 GBC, 1 AVC)	More (*TP53*) or less (*NRAS, KRAS, ERBB215, PIK3CA*) frequently mutated genes were identified	This is another study confirming the potential utility for umbrella studies	[124]
41 (Diffusely infiltrating type CCA; 24 ERCP bile samples, 17 tumor samples)	tNGS on bile samples was feasible and comparable to tumor tNGS Diffusely infiltrating type CCA was genetically distinct from mass-forming type CCA	Encouraging results provide ground for larger studies to evaluate the reliability of TS on bile samples	[125]
41 (32 ICC, 9 GBC, WES in 2)	Comparison of ICC with GBC revealed these two types are genetically distinct	Further investigation of chromatin remodeling could lead to the development of novel therapies	[126]
75 (55 ICC, 20 ECC; 26 surgical resections, 49 biopsies)	Genetic aberrations were significantly different between ICC and ECC	TS could identify mutated genes-based subgroups of patients with potential prognostic and therapeutic relevance	[127]
153 (70 ICC, 57 ECC, 26 GBC)	*IDH1/2* and *BAP1* mutations were characteristic of ICC, while *KRAS* and *TP53* were more frequent in ECC and GBC Potentially actionable mutations were identified in 104/153 (68%)	Clinical utility of molecular classification identified by this study requires evaluation by clinical trials	[128]
554 (412 ICCs, 57 ECCs and 85 GBCs)	Most frequently mutated genes: ICC: *TP53, CDKN2A/B, KRAS, ARID1A, IDH1* ECC: *KRAS, TP53, CDKN2A/B, SMAD4* GBC: *TP53, CDKN2A/B, ARID1A, ERBB2*	In the ICC group, *TP53, KRAS* and *FGFR2* mutations can be used as prognostic markers Identification of FGFR mutations in ICC patients could predict therapeutic response to *FGFR* inhibitors (BGJ398, pazopanib, dovitinib, TAS-120) tNGS can be utilized for clinical benefit and for designing umbrella and basket studies	[129]

Cholangiocarcinoma (CCA); extrahepatic cholangiocarcinoma (ECC); gallbladder cancer (GBC); intrahepatic cholangiocarcinoma (ICC); whole-exome sequencing (WES); targeted sequencing (TS).

**Table 7:** Targeted next-generation sequencing studies on intraductal papillary mucinous neoplasms of the pancreas and pancreatic ductal adenocarcinomas.

*N*	Findings	Clinical Implications	Reference
11 (oncocytic subtype IPMN; 11 TS, 2 WGS)	Typical oncocytic subtype IPMNs did not have *KRAS* or *GNAS* mutations and only one had both *RNF43* and *PIK3R1* mutations; *ARHGAP26, ASXL1, EPHA8*, and *ERBB4* genes were mutated in more than one sample	Larger studies are required to explore the genetic profile and biologic behavior of the oncocytic subtype of IPMN	[130]
23 (IPMN)	Identification of distinct mechanisms for the development of cancer in patients with IPMN using tNGS	Potential stratification and surveillance of patients based on the risk for pancreatic cancer	[131]
TS on FNA samples from 29 pts (25 PDA, 4 AVC)	Most frequent mutations identified: *KRAS* (93%), *TP53* (72%), *SMAD4* (31%), and GNAS (10%) Feasibility, reliability and concordance of FNA as compared to tumor samples for tNGS analysis	FNA-based tNGS analysis enables biomarker-based patient selection for clinical trials	[132]
30 (PDA)	Substantial mutational heterogeneity (73%)	tNGS shapes the development of targeted therapy for pancreatic cancer	[133]
52 (48 IPMNs, 4 ITPNs)	*GNAS* was mutated in 38/48 (79%) IPMNs, KRAS in 24/48 (50%) both in 18/48 (37.5%); Other mutations were less frequent	Identification of mutations in cyst fluid could enhance diagnosis and prognostic stratification of pancreatic cystic neoplasms	[134]
76 (PDA)	22 candidate cases have been identified (14 *KRAS* wild-type, 5 *HER2* amplifications, 2 mutations in *BRCA2* and 1 *ATM* mutation)	The availability of drugs targeting these mutated or amplified genes (cetuximab, transtuzumab) enables basket design of clinical trials	[135]

Fine-needle aspiration (FNA); intraductal papillary mucinous neoplasm (IPMN); intraductal tubulopapillary neoplasm (ITPN); pancreatic ductal adenocarcinoma (PDA); patients (pts); targeted sequencing (TS); whole-genome sequencing (WGS).

**Table 8:** Whole-exome sequencing studies on hepatobiliary and pancreatic cancers.

Cancer Type	*N*	Findings	Clinical Implications	Reference
HB	6	21 mutated genes, including mutations in the WNT pathway	Larger studies are required to explore the mutational background of HB	[141]
HCC (HBV positive)	10 (110 samples, including PVTTs and intrahepatic metastases)	*ARID1A* was mutated in 14 of 110 samples (13%) *ARID1A* loss-of-function mutations may be crucial for HCC invasion and metastasis	*ARID1A* is a potential novel biomarker for treatment and prognosis	[142] †
NAFLD-related HCC	10 (11 samples, WES, TS, CNV studies)	12 genes were frequently mutated including novel genes (*FGA*, *SYNE1*)	Larger studies are required to confirm the validity of novel genes	[143]
PDA (acinar differentiation)	23	Potentially targetable mutations in well-known genes (*BRCA2*, *PALB2*, *ATM*, *BAP1*, *BRAF* and *JAK1*) were identified in 1/3 of patients	This study supports the conduction of umbrella studies	[144]
HCC	24 WES (NGS); 125 CNA in total with CGH array analysis	New recurrent mutations in *ARID1A*, *RPS6KA3*, *NFE2L2* and *IRF2* Inactivation of chromatin remodelers was frequent and was associated with alcohol Wnt/b-catenin pathway promotes tumorigenesis through both oxidative stress metabolism and MAPK pathways	Association of environmental risk factors with specific gene mutations could improve screening and early diagnosis	[145]
PDA from VLTSs (≥10 years)	35 (8 WES, 27 TS)	Frequently mutated genes were identified (*KRAS*, *TP53*, *RNF43*, *CDKN2A*, and *SMAD4*) Combined WES and TS data showed no significant difference between VLTSs and patients unselected for survival	Validity of these data must be investigated by larger studies	[146]
FLC	78 (48 WES + TES, 58 whole-transcriptome, 41 SNP arrays)	Identification of 3 molecular classes: proliferation with altered mTOR pathway, inflammation with altered cytokine production genes and unannotated	Larger studies are required to confirm the validity of the developed prognostic 8-gene expression signature (*PEAR1*, *KRTAP*, *KLRD1*, *OSBPL8*, *RPL32*, *SLC26A11*, *RGS11* and *RAPGEF1*)	[147]
HCC	87	Substantial genetic heterogeneity *NFE2L2*-*KEAP1* and *MLL* pathways were recurrently mutated	Further larger WES studies are needed for completing the cancer driver genes catalog and developing individualized therapy	[148]
PDA	99 with early stage (I and II; WES and CNA)	Substantial genetic heterogeneity 8 novel mutated genes: *EPC1* and *ARID2* (chromatin modification), *ATM* (DNA damage repair), *ZIM2*, *MAP2K4*, *NALCN*, *SLC16A4* and *MAGEA6*	The novel mutated genes identified could potentially be used as therapeutic targets but validation is required by larger studies	[149]
PDA	101 (24 WES and 77 TS)	Mutated chromatin regulating genes *MLL*, *MLL2*, *MLL3*, *ARID1A* were associated with improved survival Detection of ctDNA was associated with predictable recurrence 6.5 months before occurrence	These genes may have prognostic significance and ctDNA could potentially be used as a biomarker to predict recurrence	[150]
PDA	109	Identification of multiple novel mutated genes in PDA, with select genes harboring prognostic significance KRAS mutations were observed in >90% of cases *ARID1A* was a marker of poorer outcome *RBM10* mutation was associated with longer survival *BRAF* and *PIK3CA* mutations expand the spectrum of oncogenic drivers	PDA is a complex cancer and WES can provide insight on pathogenesis, diagnosis and therapeutic management of these tumors	[151]
ICC	135 (7 fresh frozen samples, 107 FFPE, 21 FFPE mixed HCC-ICC; WES in 8, WGS in 1)	Chromosomal translocation t(10;12)(q26;q12) leads to *FGFR2*–*PPHLN1* fusion; it is successfully inhibited by a selective *FGFR2* inhibitor in vitro	Novel fusion event (*FGFR2*–*PPHLN1*) could provide therapeutic benefit Most CCA patients harbor potentially targetable molecular alterations	[152]
HCC	231 (WES and CNA)	Mutated *RB1* was a predictor of recurrence and poor survival after HCC resection	*RB1* mutations could be used as a prognostic molecular biomarker for resectable HCC	[153]
HCC	243	28% of the tumors featured genetic alterations targeted by FDA-approved drugs and 3 groups of genes were associated with risk factors: *CTNNB1* (alcohol), *TP53* (HBV) and *AXINI*	Association of environmental risk factors with specific genes provides new potential for HCC prevention and early-stage diagnosis	[154]
HCC	503 (452 WES) *	TERT alterations were identified in 68% of the patients *AXIN1* was more frequently mutated in HBV-positive and *ARID1A* in non-virus cases Druggable kinase alterations were rarely found (<2%)	Mutations in genes coding for metabolic enzymes, chromatin remodelers and mTOR pathway could provide diagnostic and therapeutic potential	[155] ‡

Comparative genomic hybridization (CGH); copy-number alteration (CNA); fibrolamellar hepatocellular carcinoma (FLC); formalin-fixed paraffin-embedded (FFPE); hepatoblastoma (HB); hepatocellular carcinoma (HCC); intrahepatic cholangiocarcinoma (ICC); pancreatic ductal adenocarcinoma (PDA); single nucleotide polymorphism (SNP); targeted sequencing (TS); very long-term survivor (VLTS); whole-exome sequencing (WES); * 22 cases of WGS are included in Table 9; † WES analysis was performed on the Illumina Genome Analyzer II platform, which is no longer available for order on the official Illumina website; ‡ WES analysis was performed on the SureSelect Human All Exon V3 or V4 platform from Agilent Technologies.

**Table 9:** Whole-genome sequencing studies on hepatobiliary and pancreatic cancers.

Cancer Type	*N*	Findings	Clinical Implications	Reference
PDA	3	*KRAS* signaling pathway was the most heavily impacted pathway	Larger WGS studies are required for assessing clinical utility	[156]
HCC with pulmonary metastasis	4	Somatic SNVs, SVs and CNAs were similar between primary and metastatic tumors	Larger studies with multiple biopsies are required to investigate similarities and differences between primary and metastatic tumors	[157]
FLC	10	Few coding, somatic mutations, no recurrent SVs Molecular differentiation from HCC	This study supports further research on the *DNAJB1*-*PRKACA* fusion protein for potential diagnostic and therapeutic clinical implementation	[158]
HCC	22 *	*TERT* alterations were identified in 68% of the patients *AXIN1* was more frequently mutated in HBV-positive and *ARID1A* in non-virus cases Druggable kinase alterations were rarely found (<2%)	Mutations in genes coding for metabolic enzymes, chromatin remodelers and mTOR pathway could provide diagnostic and therapeutic potential	[155]
HBV-related HCC	22 (WGS and RNA seq.)	Mutations, including non-coding alterations and SVs and virus integrations can create diverse transcriptomic aberrations	Integrative analysis of WGS and RNA-Seq is crucial for understanding the importance of comprehensive GA identification, shaping new diagnostic and therapeutic avenues	[159]
HCC	27 (25 HBV- or HCV-related)	In the two multicentric tumors, WGS analysis suggested origins from independent mutations Chromatin regulation genes (*ARID1A*, *ARID1B*, *ARID2*, *MLL*, *MLL3*) were mutated in approximately 50% of the tumors Frequent integration of HBV DNA in TERT locus	GAs and carcinogenesis can be influenced by the etiological background (viral hepatitis) Further elucidation on the molecular background of HCC is required to achieve significant clinical benefit	[160]
HCC	42 (WGS, WES and whole-transcriptome seq.)	More (*TP53*, *CTNNB1* and *AXIN1*) or less (*BAP1* and *IDH1*) frequent mutations and a novel deletion in *CTNNB1* were identified; *LAMA2* was a predictor of recurrence and poor survival	Identification of GAs and virus-associated genomic changes provide new predictive and therapeutic potential	[161]
HCC	88 (81 HBV positive)	HBV integration is more frequent in the tumors (86.4%) than in adjacent liver tissues (30.7%) Recurrent HBV integration in *TERT*, *MLL4* and *CCNE1* genes, with upregulated gene expression	The number of HBV integrations is associated with survival and could have prognostic significance	[162]
HCC/LCB	90 (30 LCB, 60 HCC)	LCBs feature recurrent mutations in *TERT* promoter, chromatin regulators (*BAP1*, *PBRM1* and *ARID2*), a synapse organization gene (*PCLO*), *IDH* genes and *KRAS* *KRAS* and *IDH* mutations were more frequent in hepatitis-negative LCBs and are associated with poor disease-free survival	Chronic hepatitis has a major impact on the mutational status of liver cancer	[163]
PDA	100 (WGS and CNV analysis)	Identification of altered genes (*TP53*, *SMAD4*, *CDKN2A*, *ARID1A* and *ROBO2*), novel gene mutations (*KDM6A* and *PREX2*) and frequent targetable gene mutations (*ERBB2*, *MET*, *FGFR1*, *CDK6*, *PIK3R3* and *PIK3CA*)	*KDM6A* and *PREX2* are potential biomarkers and therapeutic targets	[164]
HCC, ICC	300 (268 HCC, 24 ICC, 8 combined HCC/ICC)	Mutations related to liver carcinogenesis and recurrently mutated coding and noncoding regions were identified Known (*CDKN2A*, *CCND1*, *APC*, and *TERT*) and novel (*ASH1L*, *NCOR1*, and *MACROD2*) cancer-related genes were identified in SV analysis	WGS is crucial for detection of cancer driver genes Association of risk factors (smoking, HCV, HBV, alcohol) with specific mutations can predict tumorigenesis and provide prognostic potential	[10]

Copy number alteration (CNA); fibrolamellar carcinoma (FLC); genomic alterations (GA); hepatocellular carcinoma (HCC); intrahepatic cholangiocarcinoma (ICC); liver cancer displaying biliary phenotype (LCB); pancreatic ductal adenocarcinoma (PDA); single nucleotide variation (SNV); structural variation (SV); whole-exome sequencing (WES); whole-genome sequencing (WGS); * 452 cases of WES are included in Table 5A.
